# The substrate specificity, enantioselectivity and structure of the (*R*)-selective amine : pyruvate transaminase from *Nectria haematococca*

**DOI:** 10.1111/febs.12778

**Published:** 2014-04-07

**Authors:** Christopher Sayer, Ruben J Martinez-Torres, Nina Richter, Michail N Isupov, Helen C Hailes, Jennifer A Littlechild, John M Ward

**Affiliations:** 1 Henry Wellcome Building for Biocatalysis, College of Life and Environmental Sciences, University of ExeterEX4 4QD, UK; 2 Department of Biochemical Engineering, Advanced Centre for Biochemical Engineering, Torrington Place, University College LondonWC1E 7JE, UK; 3 Department of Chemistry, University College London20 Gordon Street, WC1H 0AJ, UK

**Keywords:** (*R*)-selective, aminotransferase, industrial biocatalysis, protein structure, substrate specificity, transaminase

## Abstract

During the last decade the use of transaminases for the production of pharmaceutical and fine chemical intermediates has attracted a great deal of attention. Transaminases are versatile biocatalysts for the efficient production of amine intermediates and many have (*S*)-enantiospecificity. Transaminases with (*R*)-specificity are needed to expand the applications of these enzymes in biocatalysis. In this work we have identified a fungal putative (*R*)-specific transaminase from the Eurotiomycetes *Nectria haematococca*, cloned a synthetic version of this gene, demonstrated (*R*)-selective deamination of several substrates including (*R*)-α-methylbenzylamine, as well as production of (*R*)-amines, and determined its crystal structure. The crystal structures of the holoenzyme and the complex with an inhibitor gabaculine offer the first detailed insight into the structural basis for substrate specificity and enantioselectivity of the industrially important class of (*R*)-selective amine : pyruvate transaminases.

**Database:**

The atomic coordinates and structure factors for the *Nectria* TAm in holoenzyme and gabaculine-bound forms have been deposited in the PDB as entries 4cmd and 4cmf respectively.

**Structured digital abstract:**

• TAm and TAm bind by x-ray crystallography (View interaction)

## Introduction

Transaminase (TAm; aminotransferase; EC 2.6.1.) enzymes catalyse the reversible transfer of an amino group from an amino substrate onto a ketone or aldehyde, producing a chiral amine in the case of a keto-acceptor 1–4. TAms are involved in the metabolism of most of the 20 naturally occurring proteinogenic amino acids and many other amine-containing compounds. TAm enzymes with sufficiently relaxed substrate range are useful tools in the synthesis of chiral amino acid analogues 5–7, e.g. *tert*-leucine 8, homophenylalanine 9, and glutamate analogues for the study of neuronal receptors 10.

TAms use the cofactor pyridoxal 5′-phosphate (PLP), the biologically active form of vitamin B6 11,12 which normally covalently binds to an active site lysine via a Schiff base (internal aldimine; Scheme6). The TAm mechanism is made up of two half-reactions. In the first half-reaction the donor substrate forms a Schiff base with the cofactor and after further intermediate steps, including an α-proton abstraction step, transfers its amino group to the cofactor (external aldimine), resulting in a keto acid and enzyme-bound pyridoxamine 5′-phosphate (PMP). In the second half-reaction the amino group is transferred from PMP to an acceptor ketone or aldehyde restoring the PLP internal aldimine and producing a chiral amino group if the acceptor was a ketone or a primary amine if the acceptor was an aldehyde. α-ketoglutarate is a common amino acceptor substrate of TAms, but some use pyruvate as an amino group acceptor and are called in such cases substrate : pyruvate TAms, e.g. serine : pyruvate TAm from *Sulfolobus solfataricus* from TAm class V 13,14.

**Figure 6:**
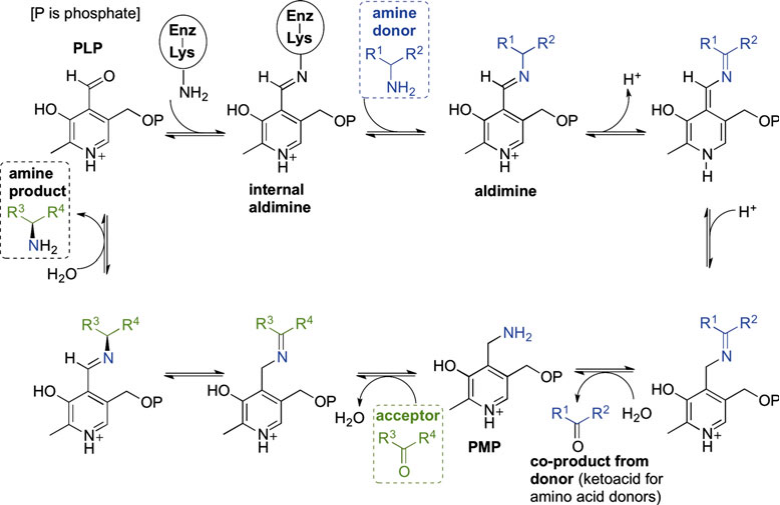
Scheme A representation of the transaminase enzyme mechanism showing key intermediates.

TAms are currently of key interest to biotechnology for both the asymmetric synthesis and resolution of chiral amines. Within the many types of TAm enzymes, specifically interesting for industry are the (*S*)- and (*R*)-selective TAms which can catalyse the transfer of an amino group from substrates containing an amino group distal to the carboxyl group (ωATms 15) or no carboxyl group (amine transaminases 16). Several of these enzymes have been biochemically characterized. In particular the ωTAms from *Vibrio fluvialis* 16 and *Chromobacterium violaceum* 17 have been studied as potential biocatalysts in the production of chiral amines in a stereoselective fashion. These enzymes all belong to the Pfam class III of transaminases 18 and have a PLP type I protein structure fold. They show activity towards (*S*)-α-methylbenzylamine [(*S*)-MBA] and have varying activities towards other amines such as β-alanine and other aliphatic amines. They also use pyruvate and many other ketones and aldehydes as the amino group acceptor 17.

To date enzymes capable of catalysing the (*R*)-selective transamination of ω-substituted amines have not been widely studied but the production of such chiral amines remains very challenging synthetically. Recently a collaboration between Codexis and Merck resulted in the production of a mutant (*R*)-specific transaminase from *Arthrobacter* sp. (ArRMut11) to aminate sterically demanding 1,3-ketoamides and generate an (*R*)-chiral amine used in the manufacture of sitagliptin, which is used in the treatment of type II diabetes 19. The mutant ArRMut11 has also been reported to transaminate bicyclic ketones such as tetralone 20,21. Most transaminases (such as the (*S*)-ωTAms) belong to PLP type I fold and are all specific towards the (*S*)-enantiomer of their substrates. Interestingly, enzymes which belong to Pfam TAm class IV and have PLP type IV fold can catalyse transamination of either d-amino acids or branched chain l-amino acids. Unlike most of the other PLP enzymes the catalysis in these enzymes occurs on the *re* side of the cofactor. Since both (*S*)- and (*R*)-enantiomers can be accommodated within one architectural fold depending on the specific sequences of the enzymes, this class of TAms potentially contains enzymes capable of transamination to give ω-substituted amines. Höhne *et al*. 22 analysed sequences of a large group of TAms of this Pfam class and found a fingerprint sequence for enzymes potentially capable of (*R*)- ωTAm activity. Several of these enzymes demonstrated high enantioselective activities towards *R*-α-methylbenzylamine [(*R*)-MBA], (2*R*)-aminohexane and (2*R*)-amino-4-phenylbutane. Several three-dimensional structures of (*S*)-specific TAms have been elucidated 23,24. Recently the crystallization of an (*R*)-TAm has been reported 25; however, to date no structure is available.

Gabaculine (5-amino-1,3-cyclohexadienylcarboxylic acid) is a common suicide inhibitor of both α- and ω-transaminases. Gabaculine binds to the TAm enzyme to form a Schiff base with the PLP cofactor. The complex then undergoes a number of bond rearrangements to form an unstable intermediate, which is spontaneously converted to *m*-carboxyphenylpyridoxamine phosphate (mCPP). This compound results in an irreversible aromatic modification of the cofactor where the Schiff base formed between gabaculine and PLP becomes a non-hydrolysable single bond 26, which binds tightly to the active sites of TAms. The formation of this complex can aid our understanding of the active site conformation adopted for determining substrate specificity and enzymatic turnover 13,24.

In this paper we describe the substrate specificity for an (*R*)-specific ωTAm from *Nectria haematococca* and its three-dimensional structures in both the PLP and gabaculine-bound forms.

## Results

### (*R*)-selective TAm design, cloning and initial assays

Previous sequence structure studies on both d-amino acid TAms (DATAs) and branched chain TAms (BCATs; class IV 14) indicated that there was a possibility that these types of TAms might be able to perform (*R*)-selective transamination. A blast search for class IV TAms was performed and several fungal branched chain enzymes were identified and aligned using clustalw (http://embnet.vital-it.ch/software/ClustalW.html) followed by the creation of an unrooted tree. From one of the subgroups of this unrooted tree, four amino acid sequences were selected and these were later shown to match TAms reported to have (*R*)-selective activity [i.e. accepting (*R*)-compounds such as (*R*)-MBA or d-alanine] 22. A detailed analysis of the retrieved sequences led us in 2009 to the identification of a TAm belonging to *N. haematococca*, also known by its asexual name *Fusarium solani* 27,28, as a new TAm whose properties had not been previously reported.

The *Nectria* TAm protein sequence was used to design a synthetic gene using optimal codons for *Escherichia coli*. The synthetic genes were designed to contain three flanking restriction sites, *Nde*I, *Hin*dIII and *Xho*I, which were used to create a wild-type (wt) and a His-tagged version of the *Nectria* TAm synthetic gene respectively (Fig. S1). The gene was cloned into the pET29a vector and overexpressed in *E. coli* in a soluble form.

**Figure 1:**
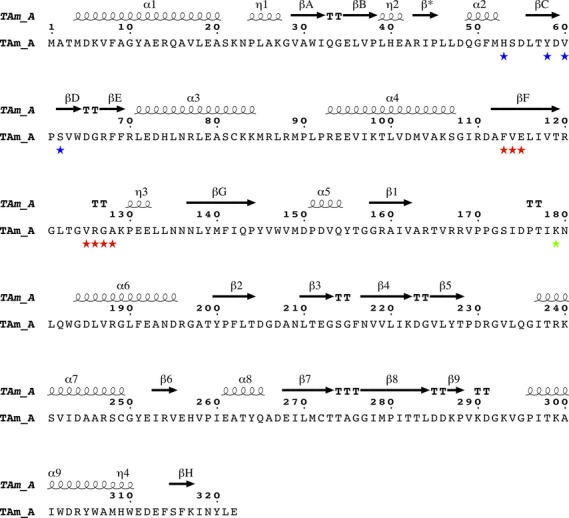
The amino acid sequence and secondary structure of the *Nectria* TAm. The residues proposed by consensus analysis to be important for (*R*)-selective amine activity are marked 22. Motif 1 (blue stars): H(53); Y(58); V(60) and S(62). Motif 2 (red stars): F(113); V(114); E(115); V(125); R(126); G(127); A(128). The active site Lys179 is highlighted by a green star. The secondary structural elements are indicated above the sequence, respectively, as α-helices, η-3_10_ helices and β-strands. The N-terminal domain β-sheet is shown as βA–βH, the PLP binding domain β-sheet as β1–β9. The β-strand forming the inter-subunit β-strand with its symmetry equivalent is marked as β*. The secondary structure assignment and the figure were produced using espript 45.

The amino acid sequence of the *Nectria* TAm is shown in Fig.1 highlighting the two motifs identified by Höhne *et al*. 22 as being responsible for (*R*)-selective transamination.

Preliminary enzyme activity from small scale cultures indicated that crude lysate from constructs pQR1022 (wt) and pQR1023 (His-tagged) produced acetophenone when using (*R*)-MBA plus pyruvate as substrate pairs (Fig. S2). However, no significant activity was noted when using (*S*)-MBA (crude lysate or resuspended pellets).

### *Nectria* TAm substrate specificity and the production of (*R*)-amines

Based on the analysis of the primary amino acid sequence and the preliminary assay data the *Nectria* TAm was believed to be (*R*)-selective. The substrate specificity and the enantioselectivity of the transaminase were then investigated in detail using a colorimetric assay previously described by Hopwood *et al*. 29 (Scheme7). The specific and relative activities of the *Nectria* TAm in the deamination of various racemic (*rac*) and single isomer amines (**1–7**) are summarized in Table1. In general, the *Nectria* TAm accepted a number of aromatic and cyclic amines with different activities. The highest specific activity was determined to be in the oxidative deamination of (*R*)-MBA (*R*)-**1**, while (*R*)-1-cyclohexylethylamine (*R*)-**2**, (*R*)-4-chloro-α-methylbenzylamine (*R*)-**3** and (*rac*)-1-methyl-3-phenylpropylamine **6** were converted with good specific activities as well (25%–62% relative activity). With the amines 1,2,3,4-tetrahydro-1-naphthylamine **4** and α-ethylbenzylamine **5** only a low activity could be detected, and no conversion of cyclohexylamine **7** to cyclohexanone was detected at all.

**Figure 7:**
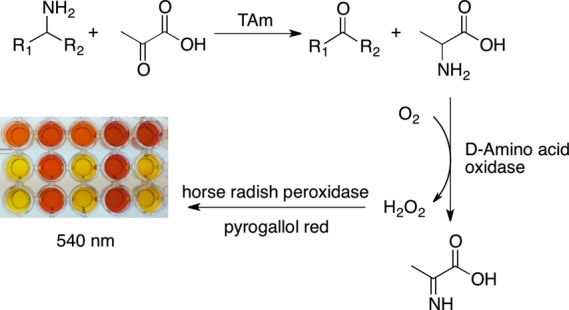
Scheme Spectophotometric assay used to screen the *Nectria* TAm with several chiral amines.

**Table 1:** Spectophotometric assay used to screen the *Nectria* TAm against several chiral amines 1–6 and amine 7 and the substrate acceptance and relative activities.

Substrate	Specific activity (U·mg^−1^)		Relative activity (%)
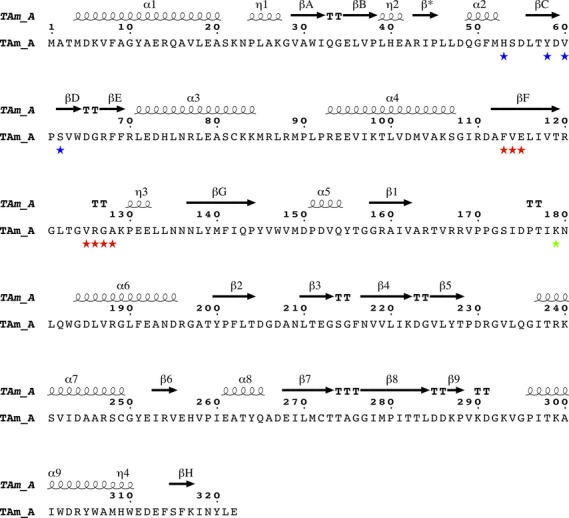 **1**	(*R*)	1.83	100
	(*S*)	0.02	0.90
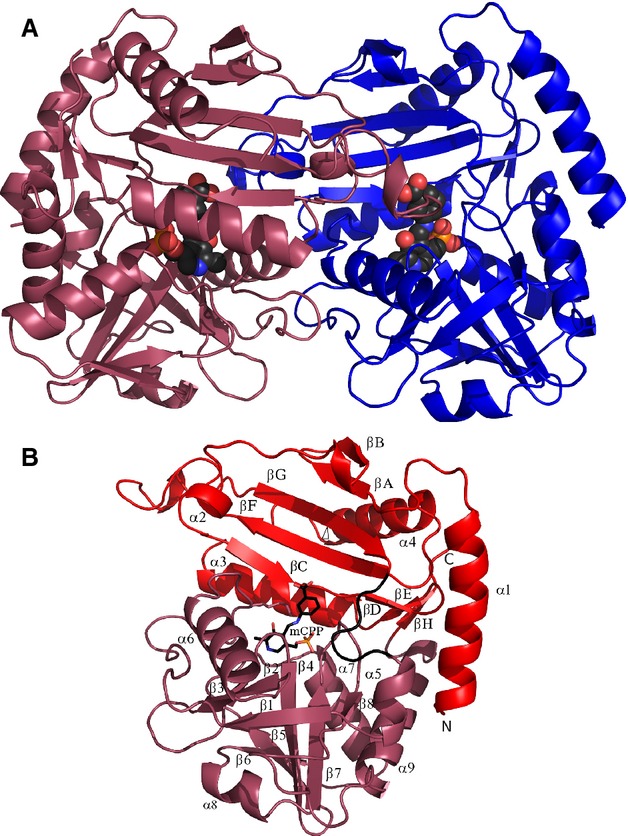 **2**	(*R*)	1.14	62
	(*S*)	0	0
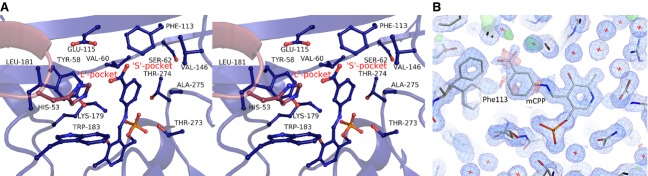 **3**	*rac*	0.63	35
	(*R*)	0.85	46
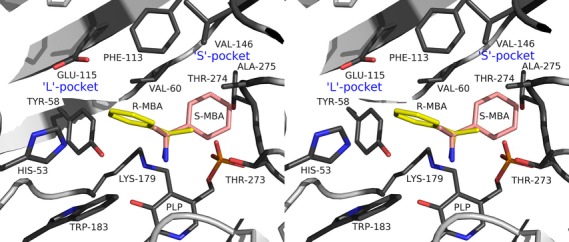 **4**	*rac*	0.02	1.1
	(*R*)	0.08	4.5
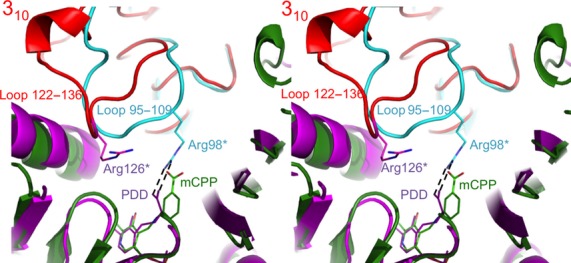 **5**	*rac*	0.16	8.6
	(*S*)	0	0.13
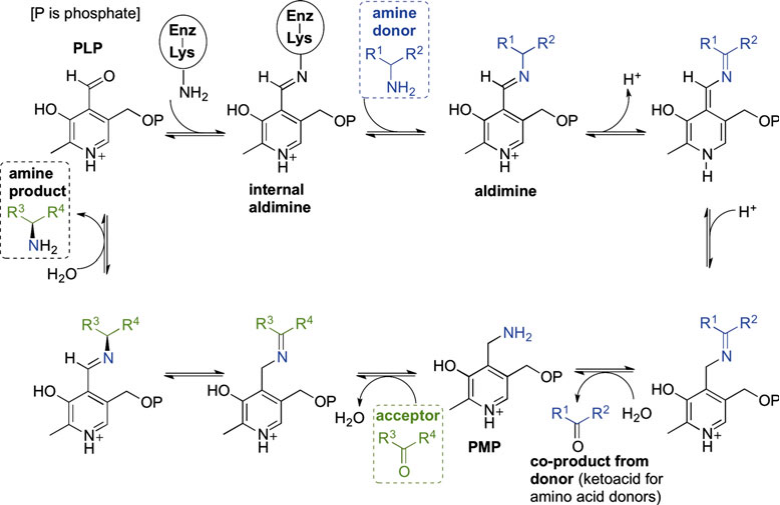 **6**	*rac*	0.46	25
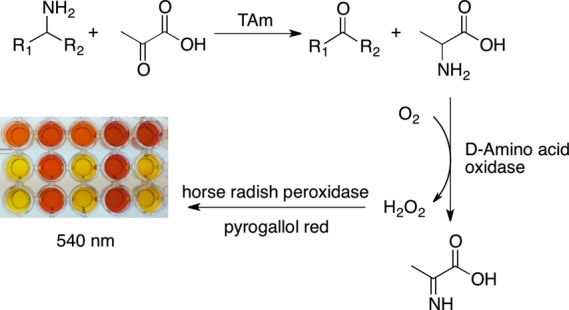 **7**	–	0	0.20

Interestingly, when considering the structures of the substrates and the specific activities obtained it seems that the position of the amino group relative to the ring is a crucial parameter. While (*R*)-**1** was converted with excellent activity, the use of (*rac*)-α-ethylbenzylamine **5** incorporating one further carbon in the side chain led to a significant decrease in activity (9% relative activity). However, a longer carbon chain between the ring and the amine, with the amine still in the α-position to a methyl group as in (*rac*)-**6**, led to decreased activity as well, but this was still approximately threefold higher than for (*rac*)-**5**. These results indicate that a methyl group α to the amine seems to be beneficial with respect to the specific activity of the *Nectria* TAm.

The results from the colorimetric assay (Scheme7, Table1) using enantiopure and racemic amines also confirmed the (*R*)-selectivity of the *Nectria* TAm. In particular, the activities observed in the conversions of **1** and **2** to the corresponding amines, where both enantiomers were available, clearly demonstrated the enzyme preference for the (*R*)-enantiomer.

To further confirm the enantiopreference of the enzyme, its selectivity in the reverse reaction (reductive amination) was also carried out, as shown in Scheme8, using lactate dehydrogenase (LDH) for pyruvate co-product removal as previously reported 21,30.

**Figure 8:**
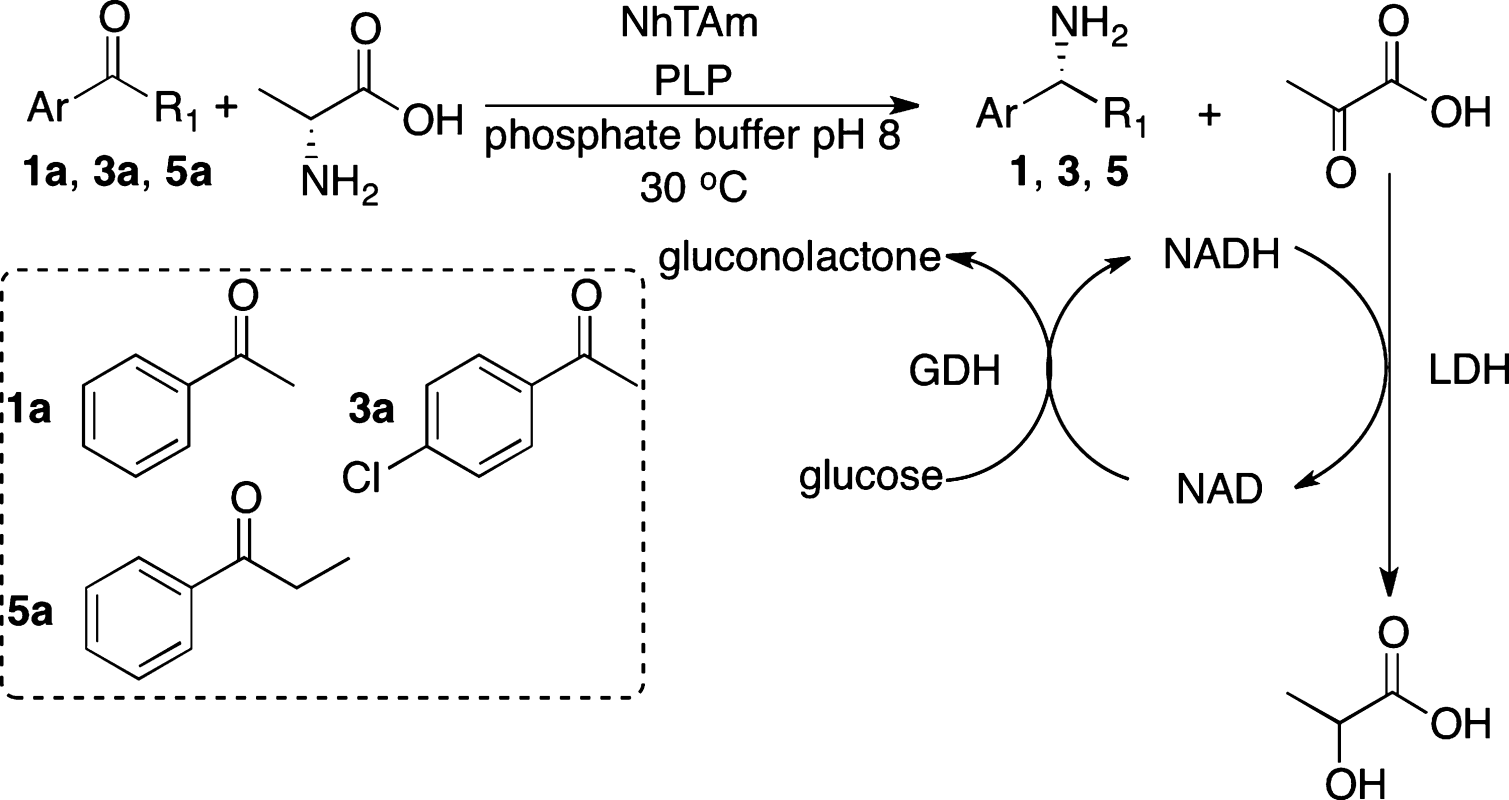
Scheme Asymmetric reductive amination of 1a, 3a, 5a catalysed by *Nectria* TAm with simultaneous co-product removal using lactate dehydrogenase (LDH) and glucose dehydrogenase (GDH) and NAD^+^ as cofactor.

The reaction products in the reductive amination of acetophenone **1a**, 4-chloracetophenone **3a** and propiophenone **5a** to **1**,**3** and **5**, respectively, were analysed by chiral HPLC. For all three ketones the (*R*)-amine was the sole reaction product indicating an enantiomeric excess > 98%, by comparison with HPLC data for the racemic and single isomer standards available (see Table S1 for chiral HPLC details). This provided additional support for the (*R*)-selectivity of the *Nectria* TAm.

### Structure of the *Nectria* TAm

The native (internal aldimine form) *Nectria* TAm structure and its complex with the inhibitor mCPP (gabaculine-PLP adduct) were solved and refined to resolutions of 1.68 and 1.50 Å respectively. The resulting *R* factors for the native and complex structures were 15.8% and 15.3% with *R*_free_ values of 18.8% and 18.0%, respectively (Table2). The *G* factors calculated for each model confirmed that the structures have acceptable stereochemical properties 31. There are no residues in the *cis* configuration.

**Table 2:** Summary of data processing and refinement statistics. Values in parentheses are given for the outer resolution shell. *R*_merge_ = Σ_*h*_ Σ_*J*_|〈*I*_*h*_〉 − *I*_*J*_(*h*)|/Σ_*h*_ Σ_*J*_*I*(*h*), where *I*(*h*) is the intensity of reflection *h*, Σ_*h*_ is the sum over all reflections and Σ_*J*_ is the sum over *J* measurements of the reflection. *R*_cryst_ = Σ||*F*_o_| − |*F*_c_||/Σ|*F*_o_|. Target values are given in square brackets. Wilson *B* factor was estimated by sfcheck 46. Ramachandran plot analysis was performed by procheck.

	Native	Gabaculine complex
Space group	P2_1_2_1_2	I222
Wavelength (Å)	0.92	0.98
Unit cell (Å)	*a *=* *106.1, *b *=* *133.9, *c *=* *61.7	*a *=* *61.7, *b *=* *100.3, *c *=* *130.3
Solvent content (%)*V*_M_ (Å^3^·Da^−1^)	59.3% (3.0)	55.8% (2.8)
Number of chains per asymmetric unit	2	1
Resolution range (Å)	29.9–1.68 (1.73–1.68)	39.85–1.5 (1.54–1.50)
Multiplicity	5.6 (5.2)	6.4 (7.3)
Unique reflections	93 564	64 789
Completeness (%)	98.8 (94.3)	99.9 (99.9)
*R*_sym_ (%)	9.6 (84.8)	9.6 (102.7)
*I*/σ(*I*)	13.9 (2.2)	16 (3.2)
Wilson *B* factor (Å^2^)	25.1	12.0
REFMAC RMS error (Å)	0.09	0.06
No. water molecules	1085	508
*R*_cryst_%	15.8	15.3
*R*_free_% (5% of total data)	18.8	18.0
Rmsd bond lengths (Å)	0.010 [0.019]	0.010 [0.019]
Rmsd bond angles (°)	1.4 [1.99]	1.4 [1.99]
Occupancy of inhibitor	N/A	1
Average *B* factor (Å^2^)		
Protein	18.2	16.9
Solvent	33.7	33.6
Gabaculine	N/A	21.9
Ramachandran plot analysis, residues (%) in		
Most favoured regions	91.8	90.9
Additional allowed regions	7.8	8.0
Generously allowed regions	0	0.7
Disallowed regions	0.4	0.4

All amino acid residues of the *Nectria* TAm were modelled into the electron density of both structures along with the first two amino acids of the C-terminal His-tag. An MES buffer molecule was modelled with occupancy 0.8 in the active site of the native enzyme.

**Figure 2:**
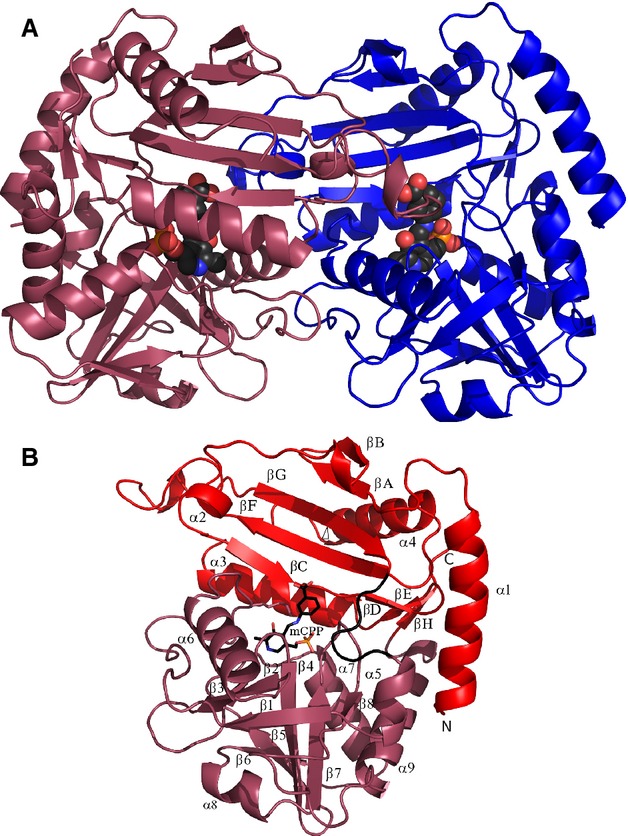
(A) A cartoon diagram of the *Nectria* TAm dimer viewed normal to its molecular dyad; the individual subunits are shown in blue and purple. The mCPP adduct is shown as a space-filling model. (B) A cartoon diagram of the *Nectria* TAm subunit with the PLP binding domain and the N-terminal domain coloured purple and red respectively. The interdomain loop region 146–151 is shown in black. The secondary structure elements are numbered, the N-terminal domain strands are labelled βA–βH, and the PLP binding domain strands β1–β8. The mCPP adduct is shown as sticks indicating the location of the active site cavity.

The protein eluted from the gel filtration column at a volume consistent with it forming a dimer in solution. The asymmetric unit of the holoenzyme contains the catalytic dimeric molecule of the *Nectria* Tam (Fig.2A). The gabaculine complex crystals contain only one subunit of TAm, with two subunits of the molecular dimer related by the crystallographic dyad. Most of the related DATAs and BCATs are dimeric, although a significant number of BCATs are hexameric, being built as a trimer of catalytic dimers.

The *Nectria* TAm belongs to the fold-type IV class of PLP-dependent enzymes and is built up of two domains of α/β type (Fig.2B). It is similar to known structures of BCATs and DATAs, with the closest structural homologue being *Thermus thermophilus* BCAT (PDB code 1WRV; 26% sequence identity). Domain 1 consists of residues 1–145 and 315–320 in an antiparallel seven-stranded β-sheet with topology −1, +4*x*, +1, −2*x*, −1, +4*x*. The second PLP binding domain consisting of residues 151–310-folds into a single eight-stranded β-sheet of mixed type with topology −1*x*, −1, +5, +1, +1, −3, −1 and direction − + + + − + −. Residues 43–45 from each subunit of the dimer form a two-stranded antiparallel β-sheet. A long N-terminal α-helix appears to be a feature of fungal (*R*)-specific amine : pyruvate TAms.

The conformations of the two subunits in the native structure are very close with rmsd of their Cα positions of only 0.13 Å over all residues. The rmsd of Cα positions between the mCPP complex structure and the native structure over all residues is only 0.21 Å. This is smaller than the rmsd between subunits within a single structure of many other PLP enzymes. This suggests that the high rigidity of this enzyme may be a consequence of the requirement to maintain a high enantioselectivity within the protein scaffold for both (*R*) and (*S*) stereoselective activities.

### Active site

The active site is formed by residues from both the N-terminal and the PLP binding domains of one subunit of the *Nectria* TAm and the N-terminal domain from the adjacent subunit. The PLP cofactor in the holoenzyme structure is bound at the bottom of the active site pocket with the *re* side of the pyridine ring exposed to solvent and the *si* side resting on the side chain of Leu234. It is covalently bound to Lys179 via a Schiff base. The PLP phosphate group is anchored by a salt bridge to the side chain of Arg77 and H-bonds with the main chain nitrogen atoms of Ile237 and Thr238 and the side chain oxygens of Thr238 and Thr274. The PLP pyridine nitrogen is coordinated by the carboxyl group of Glu212. Höhne *et al*. 22 describe two pockets for the active sites of Pfam class IV TAms with two binding sites for groups of the incoming substrates (methyl and phenyl groups in MBA) referred to as S (small) and L (large) pockets.

### mCPP adduct binding

**Figure 3:**
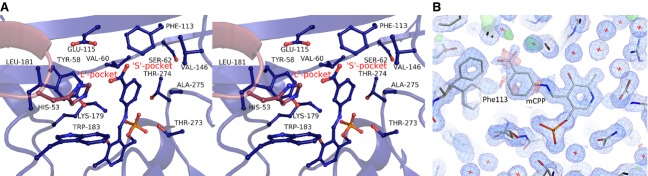
(A) A stereo diagram showing the *Nectria* TAm active site with the bound mCPP adduct. Residues forming the L and S binding pockets are shown as sticks. (B) The 2*F*_o_ − *F*_c_ electron density map (blue) was contoured at 1 σ for the gabaculine-PLP bound adduct and neighbouring residues. The positive *F*_o_ − *F*_c_ electron density map is shown in green and negative in red with both contoured at 3.1 σ. The two conformations of the Phe113 induced by the mCPP binding are shown with the disorder of the carboxyl group of mCPP highlighted by the negative *F*_o_ − *F*_c_ density.

In order to analyse the active site binding pockets adjacent to the cofactor the *Nectria* TAm was co-crystallized with the inhibitor gabaculine, which forms the mCPP adduct with PLP in the enzyme active site. The mCPP adduct is positioned centrally in the active site in close proximity (< 4.5 Å) to the S pocket outlined by the side chains of residues Phe113, Val60 and Glu115 but does not fit into it (Fig.3A). This site is responsible for binding of the methyl group of MBA. On the opposite side of the active site the L pocket outlined by residues Tyr58, Leu181, Trp183 and His53 from the adjacent subunit forms a much larger cavity which can bind the phenyl group of substrates such as (*R*)-MBA. Thus the relative positioning of the small restrictive pocket and the large pocket gives the enzyme its (*R*)-enantioselectivity towards ω-amines. The formation of the mCPP adduct causes the rotation of the PLP pyridine ring by ˜ 35° in relation to its position in the holoenzyme structure; the positions of the PLP phosphate and pyridine nitrogen remain fixed. This angle of rotation matches those observed between the holoenzyme structure and complexes in both *Bacillus* sp. DATA 32 and *E. coli* BCAT 33. The carboxyl group of the mCPP adduct is disordered due to its clash with Phe113, which is also partially displaced to a different, strained conformer not observed in the native *Nectria* TAm structure (Fig.3B).

### Determinants of the enzyme stereoselectivity

**Figure 4:**
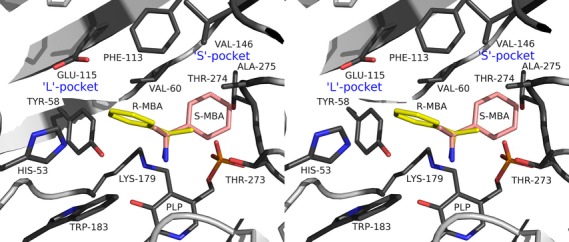
A stereo diagram showing the structural basis of the (*R*)-enantioselectivity of the *Nectria* TAm. Both enantiomers of MBA were positioned for catalysis based on the structure of the mCPP complex and the requirement for the scissile Cα-H bond to be normal to the plane of the pyridine ring of PLP. The (*S*)-MBA enantiomer in this position is pointing into the S pocket where it is sterically hindered by residues Thr273-Ala275.

To explain this enantioselectivity of the *Nectria* TAm the mCPP complex was used as a template to manually position the (*R*)-MBA-PLP external aldimine adduct in the active site for catalysis. The adduct was modelled so that orientation of the PLP pyridine ring was the same as in the mCPP complex. The (*R*)-MBA part of the adduct was positioned so that the hydrogen on the chiral carbon was normal to the plane of the PLP pyridine ring and pointing towards the NZ of the catalytic Lys179, according to the Dunathan hypothesis 34 requirement for catalysis. This positions the methyl group of MBA in the S pocket as described by Höhne *et al*. 22 and the phenyl ring in the opposite L pocket (Fig.4), where both groups fit nicely. If the (*S*)-MBA-PLP adduct is positioned for catalysis in the same manner for potential amine transfer, its phenyl group points towards the S pocket. Manual modelling of this enantiomer into the *Nectria* TAm structure revealed that the phenyl group would not fit in this S pocket due to clashes with both the side chains and main chain atoms of residues Thr273 and Thr274 and the side chain of the Ala275 (Fig.4). The limitation in space within the S pocket ensures that the *Nectria* TAm has (*R*)-amine substrate specificity.

### Substrate specificity

In the same manner in which this S pocket restricts the stereospecificity of the enzyme it also regulates the size and regioselectivity of the substrates. The enzyme shows optimal activity for (*R*)-MBA (relative activity 100%, see Table1); however, in the case of α-ethylbenzylamine when the ethyl group is positioned in the S pocket the relative activity is reduced to 9%. Modelling of this substrate into the active site reveals that when the ethyl group of α-ethylbenzylamine goes into the S pocket it is restricted by the side chain of Thr273. This would put the substrate-PLP external aldimine adduct in a less favourable position for proton abstraction by Lys179, accounting for a 10-fold reduction in activity.

In the case of the substrate (*rac*)-1-methyl-3-phenylpropylamine **6**, which has an additional carbon atom between the phenyl and amine groups compared with (*R*)-MBA, a fourfold reduction in activity was observed. Positioning this substrate (*R*-isomer) into the active site reveals that the L pocket can accommodate this larger group while the methyl group neatly fits into the S pocket as observed for MBA. Whilst this larger substrate fits in the L pocket, its movement is significantly more restricted than that observed for (*R*)-MBA with the phenyl group being positioned close to the edge of the pocket and sandwiched between the side chains of Phe113, Glu115, Tyr58, Val60 and His53. This steric hindrance could slow down the binding of (*R*)-1-methyl-3-phenylpropylamine to the active site and release of the subsequent product. It could also affect the attack of Lys179 onto the external aldimine adduct which would account for the fourfold reduction in activity. Generally, it appears that the S pocket is more limiting in controlling the substrate specificity compared with the L pocket.

Most (*R*)-specific amine TAms listed in Höhne *et al*. 22, some of which have sequence similarity to the *Nectria* TAm, have high activity towards pyruvate as an acceptor but are inert towards the acceptor α-ketoglutarate or the amino donor glutamate. We infer from this sequence similarity that the *Nectria* TAm does not have this activity. It would appear that the methyl group of pyruvate or of the reaction product d-alanine fits into the S pocket, with the carboxyl group going into the L pocket. For the α-ketoglutarate to bind in a catalytic position its α-carboxyl group should fit into the S pocket, which is not feasible due to steric restrictions.

### Relationship between the (*R*)-specific amine TAm signature motifs and its structure

While the main scaffold of Pfam class IV TAms is similar between BCATs, DATAs and (*R*)-selective amine : pyruvate TAms, different residues in specific positions enable the required substrate activities. The BCATs transfer the amino group of branched chain l-amino acids onto the α-ketoglutarate acceptor to produce the corresponding ketoacid and l-glutamate. The DATAs transfer the amino group from d-amino acids onto the α-ketoglutarate to produce the ketoacid and d-glutamate. The (*R*)-selective amine : pyruvate TAms transfer the substrate amino group onto pyruvate to produce the ketone/aldehyde and d-alanine. Höhne *et al*. 22 identified two signature motifs which allow the (*R*)-specific amine : pyruvate TAms to be distinguished from a multitude of sequence related DATAs and BCATs. These motifs are highlighted in Fig.1 for the *Nectria* TAm sequence.

The enzymatic mechanisms of both the *Bacillus* DATA 32 and the *E. coli* BCAT 33 have been thoroughly investigated and many structures of enzyme complexes containing reaction intermediates are available. The comparison of the *Nectria* TAm with structures of these other enzymes allows a greater understanding of the relationship between their sequence and enzyme activities. The conserved residues His53, Tyr58 and Val60 from the *Nectria* TAm from the first sequence motif and Phe113 from the second motif contribute to the formation of the L and S substrate pockets conferring specificity for the (*R*)-amine binding. The pair of residues, Ser62 from the first signature motif and Glu115 from the second motif, rule out the BCAT activity for the *Nectria* TAm enzyme. In the BCATs the equivalent residues Arg40 and Arg97 are involved in binding the substrates α-carboxyl and the γ-carboxyl groups respectively.

**Figure 5:**
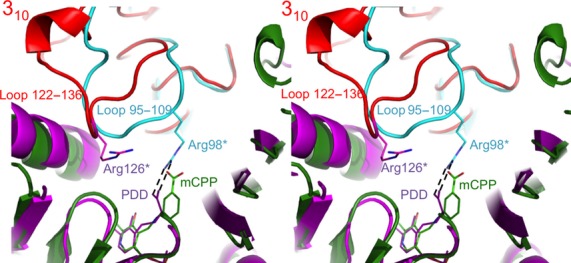
A stereo diagram showing the superposition of the dimeric *Nectria* TAm structure (subunits are shown as green and red cartoons) with the *Bacillus* sp. DATA structure (magenta and cyan cartoons). This is viewed from the solvent region into the active site cavity. The Arg98 binds the α-carboxyl group of the substrate in DATA as shown for the complex with the reaction intermediate analogue pyridoxyl-d-alanine (PDD; PDB code 3DAA). In BCAT this arginine is not conserved, although the equivalent loop retains similar conformation (not shown). In the *Nectria* TAm the corresponding loop 122–136, which contains four residues of the second fingerprint sequence motif, accepts a distinct conformation with part of it folding into 3_10_ helix. This results in significant displacement of conserved Arg126, which is unable to bind the substrate analogue mCPP carboxyl group since it is too remote. The ligands and the residues are shown as stick models.

The loop 122–135 in *Nectria* TAm which contains the four residues Val125, Arg126, Gly127 and Ala128 of the second signature motif folds into a 3_10_ helix. The conformation of the loop is significantly different from that observed for the same regions of BCAT and DATA (Fig.5). This results in a displacement of Arg126 away from the active site in relation to the equivalent Arg98 of DATA which binds the α-carboxyl group of d-alanine. Inability of the Arg126 of *Nectria* TAm to bind the substrate carboxyl group is one of the reasons for its negligible DATA activity 22.

Amongst TAm enzymes, carboxylated substrates are typically coordinated by one or two arginine residues. In the PLP fold I type TAms these arginines are highly conserved. However, in the PLP fold type IV enzymes containing BCATs, DATAs and (*R*)-amine : pyruvate TAms, the arginine residues which could bind the substrate carboxyl groups are not conserved between the different enzyme activities, with the *Nectria* TAm enzyme having no arginines in the active site. It appears that the *N. haemotococca* enzyme has no fixed carboxyl binding position for amino acids, as was earlier predicted by Höhne *et al*. 22. Thus the signature sequences for the (*R*)-selective amine TAms appear to specify a narrow S pocket, a hydrophobic L pocket and the absence of charged carboxyl binding residues thereby favouring binding of aliphatic and aromatic amines and ketones.

## Conclusion

In this work we have identified a fungal (*R*)-specific TAm from the Eurotiomycetes *N. haematococca*, and the protein sequence has been used to design a wild-type and a His-tagged version of the synthetic gene using optimal codons for *E. coli*. Cloning and expression gave an ωTAm that demonstrated (*R*)-enantioselectivity towards (*R*)-MBA (R)-**1**. The use of other chiral amines (**2**–**6**) confirmed the (*R*)-enantioselective behaviour of the *Nectria* TAm. Three ketone substrates (**1a**,**3a**,**5a**) were selected and the (*R*)-amines were formed by the *Nectria* TAm with simultaneous co-product removal using LDH and glucose dehydrogenase and NAD^+^ as a cofactor. The crystal structures of the holoenzyme of this (*R*)-selective TAm from *N. haematococca* and the complex with an inhibitor gabaculine were determined, which offers a detailed insight into the structural basis for substrate specificity and enantioselectivity. Notably, the side chains of Phe113, Val60 and Glu115 line an S pocket, and on the opposite side of the active site the L pocket is lined by residues Tyr58, Leu181, His53 and Trp183. Furthermore modelling of substrates **1**,**5** and **6** in the active site of the TAm was able to rationalise the relative specific activities and enantioselectivities that have been observed for this enzyme. This work highlights how the use of detailed structural information can help to understand the enantioselectivity and substrate specificity of this *Nectria* TAm enzyme. The information obtained will aid the use of rationally directed mutagenesis of this and other industrially important transaminases.

## Materials and methods

### Gene design, cloning and expression of *Nectria* TAm

A blast search 35 was used to screen for class IV TAms and the *Nectria* TAm protein was selected. The protein sequence was used to design a synthetic gene using the dna 2.0 software optimized for expression in *E. coli* and synthesized by dna 2.0 (Menlo Park, CA, USA). The two restriction digested versions of the gene (*Nde*I–*Hin*dIII and *Nde*I–*Xho*I) were then ligated into the expression vector pET29a^+^ and transformed into BL21(DE3) pLysS, creating the pQR1022 (wt) and pQR1023 (6x-His-tag) constructs respectively (Fig. S1).

### Preliminary expression of the Nectria TAm for biochemical assays

A 5 mL overnight culture of the BL21(DE3) pLysS containing pQR1022 (no His tag) was grown in tryptone-yeast (TY) medium for 16 h at 37 °C, 220 r.p.m. The non-His-tagged version was used initially to assess enzyme activity in case the His-tag inhibited activity. The His-tag was found to have no effect on activity and later purification and work was carried out with the His-tagged enzyme. To initiate the overexpression, the overnight cultures were diluted 1 : 100 into 20 mL of TY fresh medium containing both kanamycin (0.05 mg·mL^−1^) and chloramphenicol (0.03 mg·mL^−1^) and incubated until an *A*_600 nm_ of 0.7–0.8 was reached at 37 °C, 220 r.p.m. Then isopropyl thio-β-d-galactoside (IPTG; 0.5 mm) was added and the culture was left for a further 5 h at 30 °C, 220 r.p.m. Cells were harvested after 5 h and centrifuged for 10 min at 14 000 ***g***; the cell pellet was then re-suspended in lysis buffer (100 mm HEPES and 0.4 mm PLP, pH 8) and sonicated using a 10 s per 10 s (on/off) protocol at 12 μm. The resulting material was centrifuged and separated into crude lysate or resuspended pellet and stored at −20 °C.

### Optimized expression for enzyme characterization

A 5 mL overnight culture of the BL21(DE3) pLysS containing pQR1023 (6x-His-tag) was grown in LB medium containing kanamycin (0.05 mg·mL^−1^) for 16 h at 37 °C, 220 r.p.m. The overnight cultures were used to inoculate fresh LB medium (20 mL) containing kanamycin (0.05 mg·mL^−1^) to give a final 0.05 *A*_600 nm_ of 0.05. Cultures were grown at 37 °C and 120 r.p.m. until an *A*_600 nm_ of 0.7–0.8 was reached, IPTG (0.5 mm) was added and the temperature was reduced to 25 °C and the cultures incubated for 20 h. The cells were harvested by centrifugation for 10 min at 6000 ***g*** and stored at −20 °C until further use. For the disruption of the cells a 10% (w/v) solution was prepared using an equilibration buffer (100 mm HEPES pH8, containing 0.5 mm PLP and 20 mm imidazole). The cells were disrupted by sonication (10 × 15 s on ice, 0.10 output), and after each cycle the cells were kept on ice for 1 min. The resulting material was centrifuged at 13 000 r.p.m., and the supernatant was used in the purification procedure. For purification the crude extract was loaded onto a column (PD10; GE Healthcare, Little Chalfont, UK) containing 2 mL of Ni-nitrilotriacetic acid material. After binding, the column was washed with three volumes of equilibration buffer, and eluted with the same buffer but containing 200 mm imidazole. For desalting a PD10 desalting column (GE Healthcare) was used and 10 mm HEPES buffer at pH 8. This purification protocol provides enzyme preparations that are optimal for biocatalysis.

### Overexpression and purification of the *Nectria* TAm for crystallization

Plasmid pQR1023 was transformed into *E. coli* BL21(DE3) pLysS and cultured overnight in LB medium containing kanamycin (0.05 mg·mL^−1^). ZYM-5052 36 medium (500 mL) was inoculated using the overnight cultures and incubated at 20 °C for 2 days with shaking at 250 r.p.m. The cells were then harvested at 4700 ***g*** and 4 °C for 20 min. The pellet was re-suspended in 50 mm Tris/HCl pH 7.5. Cells were disrupted by sonication at 10 μm (Soniprep 150; MSE (UK) Ltd, Lower Sydenham, UK) on ice for 2 min and the cell debris was removed by centrifugation at 15 000 ***g*** and 4 °C for 30 min.

The protein was purified using the 6x-His-tag using a 1 mL His-Trap FF crude column (GE Healthcare) using a gradient up to 500 mm imidazole in 50 mm Tris/HCl pH 7.5. The enzyme was then applied to a Superdex 200 HiLoad 16/60 gel filtration column (column volume 120 mL) and was eluted with 1 column volume in 50 mm Tris/HCl, 0.1 m NaCl pH 7.5 at 1.0 mL·min^−1^. This purification protocol gives enzyme material that can readily crystallize (see section on Crystallization).

### Determination of the *Nectria* TAm stereoselectivity using the acetophenone production assay

(*R*)- or (*S*)-selectivity of crude lysates/resuspended pellets from pQR1022 and pQR1023 were tested using 5 mm of the substrate pairs (*R*)- or (*S*)-MBA and pyruvate. Reactions were carried out in 1 mL reaction buffer (100 mm HEPES buffer, 0.4 mm PLP, pH 8), where 20% of that final volume corresponded to either crude lysate or resuspended pellet from pQR1022 or pQR1023, respectively. Sample reactions were taken and quenched after 1, 2 and 17 h by mixing 1 : 1 (v/v) of reaction mix with 0.2% trifluoroacetic acid solution. Production of acetophenone was followed using an HPLC C18 column at 210 nm. (*R*)- or (*S*)-selectivity of the enzyme was assessed by the production of acetophenone from (*R*)- or (*S*)-MBA.

### Determination of TAm substrate specificity

The transaminase activity of the *Nectria* TAm against selected substrates **1**–**7** was determined using the spectrophotometric assay previously described by Hopwood *et al*. 29. A volume of 10 μL of the purified *Nectria* TAm (pQR1023) was added to 190 μL of assay solution in a microtitre plate. The assay solution contained substrate (5 mm), sodium pyruvate (0.2 mg·mL^−1^), pyrogallol red (0.1 mg·mL^−1^), PLP (0.05 mg·mL^−1^), amino acid oxidase (1.2 U·mL^−1^) and horseradish peroxidise (12.5 U·mL^−1^) in sodium phosphate buffer (100 mm, pH 8). Absorbance at 510 nm was followed over a period of 30 min, and the linear slope was used to determine initial rates of the enzyme activity using the extinction coefficient (19.533 mm).

### Stereoselective formation of chiral amines from acetophenone

The stereoselectivity of the *Nectria* TAm in the asymmetric amination of different ketones was performed with simultaneous co-product removal to shift the equilibrium of the TAm reaction towards the desired amine product. Purified TAm (0.2 mg) was added to a sodium phosphate buffer solution (1 mL, 100 mm, pH 8) containing the substrate (20 mm), the co-substrates d-alanine (100 mm), β-d-glucose (60 mm), the cofactors PLP (1 mm), NAD^+^ (0.5 mm) and the co-product removal system (lactate dehydrogenase and glucose dehydrogenase, both 5 U·mL^−1^). The reaction was incubated at 30 °C and 800 r.p.m in an Eppendorf Thermomixer (Hamburg, Germany). After 72 h the reaction was stopped by the addition of 1 mL of acetonitrile containing 0.1% trifluoroacetic acid and the protein was removed by centrifugation (5 min, 13 000 ***g***). The enantiomeric excesses were determined (within the limits of detection) by analysis of the supernatants by HPLC (Agilent, Santa Clara, CA, USA) equipped with a CROWNPAK® CR(+) column (150 × 4 mm × 5 μm; Daicel, Illkirch, France) with UV detection at 254 nm. The conditions used for the detection of the different amines are given in Table S1.

### Crystallization

The *Nectria* TAm was concentrated to 10 mg·mL^−1^ using a 10 kDa membrane Vivaspin (Vivascience, MA, USA) and microbatch crystallization trials were set up using an Oryx 6 crystallization robot (Douglas Instruments, Hungerford, Berks, UK) using the JCSG+ and Morpheus screens (Molecular Dimensions, Newmarket, Suffolk, UK). The droplet contained a 50 : 50 ratio of protein solution to precipitant ratio and was covered with a 50 : 50 mix of silicon and paraffin oils.

Crystals of the holoenzyme were grown in condition D1 from the Morpheus screen containing 30% PEG 550MME and PEG 20K. Protein prepared in the presence of 5 mm gabaculine was co-crystallized in 0.1 m MgCl_2_, 50 mm Na HEPES pH 7.5 and 15% v/v PEG 400.

Data were collected on beamlines I04 and I04-1 at the Diamond Synchrotron light source (Oxford, UK) under cryo conditions (100 K in a stream of gaseous nitrogen). Data were processed using xds 37 in the Xia2 38 pipeline. All further data and model manipulation was carried out using the ccp4 suite of programs 39.

### Data collection and structure determination

The structure of holoenzyme was solved by molecular replacement (MR) using the program molrep 40. The functional dimer of *T. thermophilus* BCAT (PDB code 1WRV) was modified to match the *Nectria* TAm sequence and was used as a search model. The rotation function was calculated at 3 Å resolution and the translation search was conducted at 4 Å resolution. The rotation solution peak height was 6.6 σ and the correlation of translational solution was 0.183. The resulting MR solution was subjected to rigid body and subsequent restrained refinement in refmac5 41. The refined model was submitted to automated refinement using the arp/warp version 7.0.1 42. The model was manually rebuilt in coot 43 and refined with refmac5. The gabaculine complex, which crystallized in a different space group, was solved by MR using the coordinates of the refined holoenzyme structure. The statistics of the data processing and refinement are given in Table2. After refinement the quality of the model was checked using the program procheck 31. Images were created using the molecular graphics program pymol 44.

## Abbreviations

BCAT: branched chain transaminase
DATA: d-amino acid transaminase
L: large
LDH: lactate dehydrogenase
(*R*)-MBA: (*R*)-α-methylbenzylamine
(*S*)-MBA: (*S*)-α-methylbenzylamine
mCPP: *m*-carboxyphenylpyridoxamine phosphate
MR: molecular replacement
PEG: poly(ethylene glycol)
PLP: pyridoxal 5′-phosphate
PMP: pyridoxamine 5′-phosphate
S: small
TAm: transaminase
ωTAm: ω-transaminase
TY: tryptone-yeast extract
wt: wild-type

## Supporting Information

Fig S1 Representation of synthetic gene design.

Fig S2 Enzyme activity of the *Nectria* TAm synthetic genes.

Table S1 Chiral HPLC details for chiral amines prepared using *Nectria* TAm.
