# Supplementary material for: The substrate specificity, enantioselectivity and structure of the (R)-selective amine : pyruvate transaminase from Nectria haematococca
Source: FEBS J. 2014 Apr 7;281(9):2240–53. doi: 10.1111/febs.12778 (PMC4255305; doi:10.1111/febs.12778)
Supplement: Table S1 — Chiral HPLC details for chiral amines prepared using Nectria TAm. [file febs0281-2240-sd1.zip › febs12778-sup-0001-FigS1-S2-TableS1.pdf]

the FEBS Journal
www.febsjournal.org
Wiley Blackwell

The substrate specificity, enantioselectivity and structure of the (*R*)-selective amine : pyruvate transaminase from *Nectria haematococca*

Christopher Sayer, Ruben J. Martinez-Torres, Nina Richter,
Michail N. Isupov, Helen C.Hailes, Jennifer A. Littlechild and John M. Ward

DOI: 10.1111/febs.12778

Supporting Information

The substrate specificity, enantioselectivity and structure of the (*R*)-selective amine:pyruvate transaminase from *Nectria haematococca*

Chris Sayer, Ruben J. Martinez-Torres, Nina Richter, Michail N. Isupov, Helen C. Hailes, Jennifer Littlechild and John M. Ward

Contents

Representation of synthetic gene design: Figure S1 S2

Enzyme activity of the *Nectria* TAm synthetic genes: Figure S2 S2

Chiral HPLC details for chiral amines prepared using *Nectria* TAm: Table S1 S3

Representation of synthetic gene design

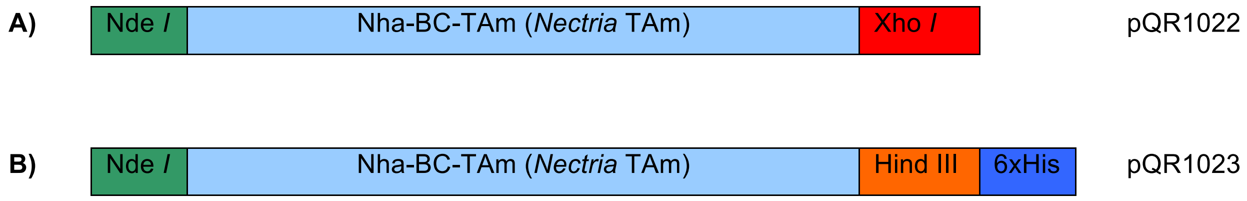

Figure S1: Representation of the synthetic gene design cloned into pET29a+ (not to scale). **A**) Wild-type protein sequence; **B**) 6x-Histag version of the synthetic gene

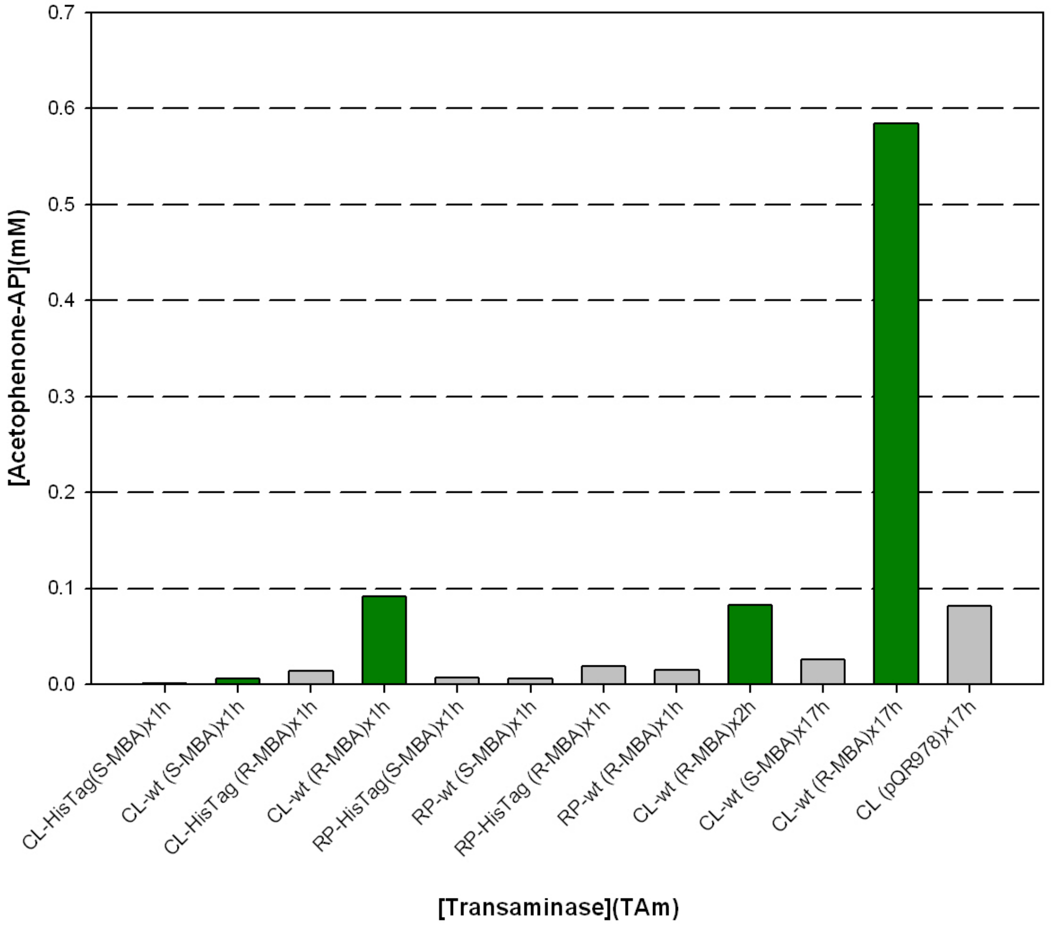

Figure S2. Enzyme activity of the *Nectria* TAm synthetic genes. Green bars correspond to the crude lysate from pQR1022 (wt). The three samples taken after 1 h, 2 h and 17 h of reaction showed a clear increase in the amount of acetophenone produced, indicating that this particular enzyme is an (*R*)-selective transaminase. pQR958 has the Pp3718 transaminase from *Pseudomonas putida* as a control.

Chiral HPLC analysis details for chiral amines prepared using *Nectria* TAm

The enantiomeric excesses were determined by analysis of the supernatants by HPLC (Agilent) equipped with a CROWNPAK®CR(+) column (Dancil, 150 mm x 4 mm x 5 μm) with UV detection at 254 nm.

Table S1: Conditions for chiral HPLC analysis

compound	detection (nm)	flow (mL/min)	solvent system	retention time (min)	Ee
α-methylbenzylamine **1**	254	0.6	10 % MeOH<br>90 % H_2O + $HClO_4$ pH 1	(*R*): 8.7<br>(*S*): 7.3	>98%
4-chloro-α-methylbenzylamine **3**	254	1.2	10 % MeOH<br>90 % H_2O + $HClO_4$ pH 1	(*R*): 14.2<br>(*S*): 12.0	>98%
α-ethylbenzylamine **5**	254	1.2	10 % MeOH<br>86 % H_2O + $HClO_4$ pH 1	(*R*): 9.8<br>(*S*): 7.2	>98%
